# Frequency of Group A *Streptococcus* Infection and Analysis of Antibiotic Use in Patients with Pharyngitis—A Retrospective, Multicenter Study

**DOI:** 10.3390/pathogens13100846

**Published:** 2024-09-28

**Authors:** Martyna Biała, Mateusz Babicki, Wojciech Malchrzak, Sandra Janiak, Dominik Gajowiak, Alan Żak, Karolina Kłoda, Piotr Gibas, Justyna Ledwoch, Anna Myśliwiec, Daria Kopyt, Anna Węgrzyn, Brygida Knysz, Patrycja Leśnik

**Affiliations:** 1 Department of Infectious Diseases, Liver Diseases and Acquired Immune Deficiences, Wroclaw Medical University, 51-149 Wroclaw, Poland; 2 Department of Family Medicine, Faculty of Medicine, Wroclaw Medical University, 50-367 Wroclaw, Poland; ma.babicki@gmail.com (M.B.);; 3 Department of Family Medicine, Nicolaus Copernicus University in Torun, Collegium Medicum in Bydgoszcz, 85-094 Bydgoszcz, Poland; 4 Indywidualna Praktyka Lekarska Dominik Gajowiak, 62-420 Radlowo, Poland; 5 Centrum Medyczne AD-Med. ul. Syrokomli 1, 51-141 Wroclaw, Poland; 6 MEDFIT Karolina Kłoda, ul. Narutowicza 13E/11, 70-240 Szczecin, Poland; 7 NZOZ NOWA-MED., 34-100 Wadowice, Poland; mygibas@gmail.com; 8 NZOZ Kraków Południe, 30-315 Kraków, Poland; justyna.ledwoch@gmail.com; 9 SZPZLO Warszawa-Wawer, 04-564 Warszawa, Poland; 10 NZOZ Przychodnia Wassowskiego, 80-225 Gdańsk, Poland; 11 Poradnia Medycyny Rodzinnej S.C. NZOZ Łapanów 186, 32-740 Łapanów, Poland; 12 Department of Microbiology, Wroclaw Medical University, 50-368 Wroclaw, Poland

**Keywords:** *Streptococcus pyogenes*, GAS, infection, antibiotics

## Abstract

*Streptococcus pyogenes* is responsible for 20–30% of pharyngitis in children and 5–15% in adults. The ineffective treatment of group A Streptococcus (GAS) infections can result in postinfectious sequelae. This study aims to evaluate the frequency of GAS pharyngitis and assess the management of patients with pharyngitis and antibiotic use. We conducted a multicenter, retrospective analysis of medical records from nine primary care centers in Poland. The study enrolled 1949 medical records of patients (children 67.4%, adults 32.6%). An infection of *Streptococcus pyogenes*, based on a rapid strep test, was diagnosed in 830 patients (42.6%). In the comprehensive study group of 1949 patients, 1054 (54.1%) were given antibiotics. Notably, 224 patients had a negative rapid strep test result but still received antibiotic treatment, underscoring the complexity of treatment decisions. The most commonly used antibiotics were oral penicillin V in 431 cases (41%) and amoxicillin in 219 cases (20.8%). We observed no significant difference between positive rapid strep test results and patients’ sociodemographic data and comorbidities. The prevalence of GAS was 42.6% in the analyzed records of patients with pharyngitis, and 54.1% were prescribed antibiotics. Antibiotics were overprescribed for sore throats. Strategies are needed to promote rational antibiotic use.

## 1. Introduction

*Streptococcus pyogenes* (GAS, group A *Streptococcus*) can be carried asymptomatically in the nasopharynx and on the skin. However, it can also cause a wide range of clinical manifestations ranging from mild localized infections to life-threatening invasive infections [1]. Non-invasive GAS diseases include pharyngitis, tonsillitis, cellulitis, impetigo, and scarlet fever. Life-threatening invasive GAS disease can present, among others, as necrotizing fasciitis and toxic shock syndrome, which are associated with high morbidity and mortality. The 2022/23 winter season saw a rise in the incidence rates of both invasive and non-invasive *S. pyogenes* infections, a trend that many countries have reported [2,3,4]. The probable cause of the higher morbidity was the increased population’s susceptibility to infections due to COVID-19 pandemic restrictions. Moreover, the increased incidence of iGAS coincided with the weeks with the highest influenza virus and RSV circulation, which cause damage to the respiratory epithelium, which could also facilitate bacterial colonization, adherence, and translocation through the epithelial barrier, promoting the way for bacterial infection [4]. According to current data from the European Centre for Disease Prevention and Control (ECDC), countries in the EU/EEA are reporting iGAS cases at the levels of pre-pandemic seasons [5]. This underscores the urgent need for effective prevention and treatment strategies.

In temperate climates, GAS pharyngitis occurs most frequently in the winter and early spring [6]. It is estimated that group A *Streptococcus* is responsible for 20–30% of sore throats in children and 5–15% of sore throats in adults, and over 616 million cases of GAS pharyngitis occur annually worldwide [7,8]. The ineffective treatment of GAS infections can result in postinfectious sequelae like acute rheumatic fever and post-streptococcal glomerulonephritis. There are approximately 470.000 new cases of acute rheumatic fever and 233.000 attributable deaths yearly, disproportionately affecting populations from developing countries [9].

We can use a rapid antigen detection test (RADT) and throat cultures to diagnose GAS infection in primary care. RADT, with its quick results (within minutes) and high specificity but moderate sensitivity, is a valuable tool in our diagnostic process. However, we must exercise caution and confirm negative results with a throat culture in children and adolescents, ensuring a thorough and comprehensive diagnosis. Throat culture stands as the gold standard for diagnosing GAS pharyngitis, a proof of its reliability and accuracy in primary care settings. If RADT yields a negative result in children or adolescents with symptoms, it is important to follow up with a throat culture. Despite the longer wait time of about 24 to 48 h, this comprehensive approach ensures accurate diagnosis.

Sore throat is one of the most common reasons for visiting a primary care doctor. This is a non-specific symptom that both bacterial and viral infections can cause. Appropriate tests, including rapid antigen tests, can help determine proper treatment and limit the use of antibiotics for viral infections.

The drug of choice for treating GAS pharyngitis is oral penicillin V or amoxicillin for ten days [7]. However, the existing Polish guidelines underline that the low dosage of amoxicillin used to treat bacterial pharyngitis might generate *S. pneumoniae* resistance [10]. Despite that, amoxicillin accounts for a significant percentage of prescribed antibiotics for treating GAS pharyngitis in Poland. In patients with a penicillin allergy, macrolides, clindamycin, and first-generation cephalosporins can be used; however, first-generation cephalosporin should not be used in patients with immediate-type hypersensitivity to penicillin [7]. All isolates of group A strep bacteria are susceptible to penicillin or cephalosporins. Nevertheless, some strains of *S. pyogenes* have developed resistance to other antibiotics. *S. pyogenes* resistance to macrolides and clindamycin is well known and varies geographically and temporally [7]. Appropriate antibiotic selection is crucial to the treatment optimization of infections and reduces the risk of drug resistance. This study aims to evaluate the frequency of GAS pharyngitis and determine sociodemographic and clinical indicators associated with GAS infection. Furthermore, this analysis aims to assess the management of patients with pharyngitis and antibiotic use. The gravity of the issue is underscored by the fact that inappropriate antibiotic use can lead to treatment failure and the development of drug-resistant strains, making our work in this area of the utmost importance.

## 2. Materials and Methods

We conducted a multicenter, retrospective analysis of medical records to evaluate the frequency of GAS pharyngitis, sociodemographic and clinical indicators associated with GAS infection, and managing patients with pharyngitis in nine primary care centers in Poland. The study included data from two seasons: December 2022–March 2023 and December 2023–March 2024. The inclusion criterion was to analyze at least 75 records of pediatric and adult patients with pharyngitis in each medical center. We analyzed the results of rapid antigen tests for GAS, antibiotic use, types of antibiotics, sociodemographic data, comorbidities (diabetes, hypertension, chronic respiratory diseases, cardiovascular diseases, and acquired or congenital immunodeficiency), and vaccination status (influenza and COVID-19). 

### Statistical Analysis

For all groups, the number of cases (N), the mean (X), median (M), range (min-max), lower and upper quartile (25q–75q), and standard deviation (SD) of the parameters were calculated. The median (interquartile range [IQR]) was used for the description of non-normally distributed data. Qualitative variables were presented as absolute values and percentages (%). The normality assumption was assessed by carrying out the Shapiro–Wilk test for the groups of data. Levene’s test was carried out to assess the homogeneity of variance assumption. For qualitative parameters, the frequency of the traits or characteristics in groups was analyzed using the χ2df test with the appropriate number of degrees of freedom, df (df = (m − 1) × (n − 1), where m—number of rows, n—number of columns). The verification of the hypothesis of equality of means groups of heterogeneous variance was performed by the non-parametric Mann–Whitney U test. *p* ≤ 0.05 was considered statistically significant. All statistical analyses were performed using EPIINFO Ver. 7.2.3.1. and Statistica Ver. 13.3. 

## 3. Results

### 3.1. Characteristics of Analyzed Data

**Figure 1:**
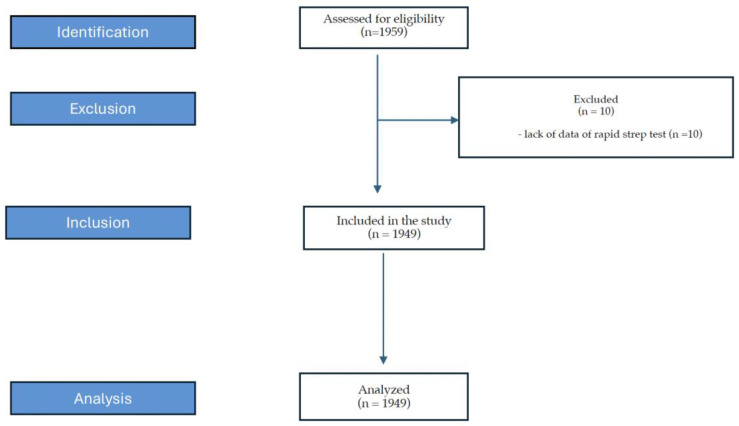
Flow chart of the analyzed records.

The study enrolled 1949 medical records of patients (Figure 1). Children’s (up to 18 years of age) medical records constituted 67.4% of analyzed data, and adults’ 32.6%. 

**Figure 2:**
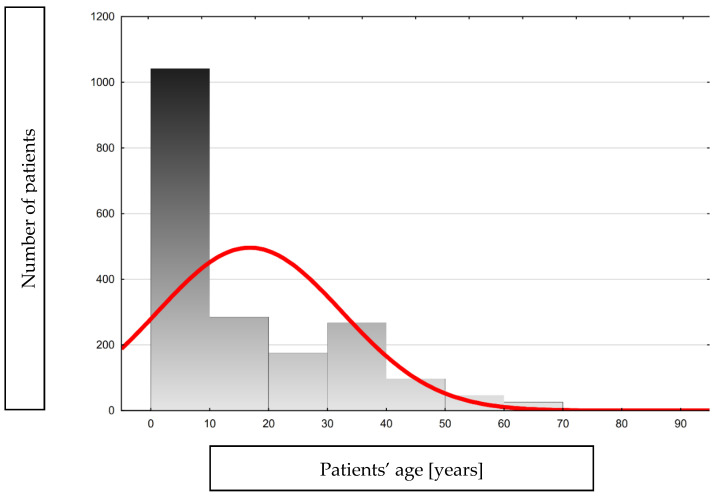
Histogram showing the age distribution of the analyzed group.

The most significant percentage of patients were from cities with a population over 500,000 (45.2%) and rural areas (25%). The remaining patients came from towns with a population of less than 500,000. In total, 56% were female and 44% male, and their age ranged from 0 to 83 years (Table 1, Figure 2). 

#### 3.1.1. Viral Infection Prophylaxis

In the study group, 128 (6.6%) patients underwent protective vaccination against the influenza virus and 556 (28.5%) patients against COVID-19.

#### 3.1.2. Comorbidity

In the analyzed group of patients, 22 (1.1%) suffer from diabetes, 104 (5.3%) have chronic respiratory diseases, 21 (1.1%) suffer from cardiovascular diseases, and 79 (4.1%) are treated for hypertension. Additionally, 12 (0.6%) patients have been diagnosed with congenital or acquired immunodeficiencies. 

### 3.2. Streptococcus Pyogenes’ Infection

An infection of *Streptococcus pyogenes*, based on rapid strep test (BIOSYNEX STREP A, Switzerland, France), was diagnosed in 830 patients (42.6%)—579 in children and 251 in adults. Median time from symptom onset to rapid strep test was 2 days (Table 2). In 312 patients’ medical records, there were no data of time from symptom onset to test.

### 3.3. Antibiotics Usage

In the comprehensive study group of 1949 patients, 1054 (54.1%) were given antibiotics, a thorough representation of the patient population. In total, 830 patients had positive strep test results. Notably, 224 patients had a negative rapid strep test result but still received antibiotic treatment, underscoring the complexity of treatment decisions. The most commonly used antibiotics were oral penicillin V in 431 cases (41%) and amoxicillin in 219 cases (20.8%). Table 3 presents the exact distribution of antibiotics used and Table 4 presents the analysis of distribution of antibiotics and rapid strep test results.

### 3.4. Statistical Analysis of Rapid Strep Test Results and Sociodemographic and Clinical Indicators

The statistical analysis showed no significant difference between positive rapid strep test results and patients’ age (*p* = 0.0577), sex (*p* = 0.809), vaccination status (influenza, *p* = 0.151; COVID-19, *p* = 0.436), and comorbidities (chronic respiratory diseases, *p* = 0.641; cardiovascular diseases, *p* = 0.676; hypertension, *p* = 0.281; and congenital or acquired immunodeficiencies, *p* = 0.948) (Table 5). Although we saw a relationship between negative rapid test results and diabetes, the difference between the groups did not meet the threshold for statistical significance (*p* = 0.0582) (Table 5).

## 4. Discussion

Our study attempted to evaluate the frequency of pharyngitis caused by *Streptococcus pyogenes* in primary care and antibiotic usage. There has been a high increase in GAS infections in the past three years. In our study group, among 1949 patients who presented with pharyngitis, 42.6% of cases were caused by *Streptococcus pyogenes* infection, confirmed by a rapid strep test. In the post-COVID period, a significant increase in infections caused by group A streptococci was observed. The prevalence of GAS infections and their complications varies between poorly developed and well-developed countries, highlighting the importance of a unified approach. In underdeveloped countries, the high number of rheumatic heart disease (RHD) and the incidence of deaths are associated with RHD. Conversely, in well-developed countries, the high incidence of deaths due to invasive GAS infection necessitates a joint effort, especially in the post-pandemic COVID-19 era. Since the beginning of December 2022, an unusually high rise in cases of and deaths from GAS infections has been reported in many European countries [4]. In Denmark, patients ≥ 85 years had the highest iGAS incidence rates, peaking at 7.4 per 100,000 in the age group per month; however, the highest relative increase compared with pre-pandemic restrictions was detected among children younger than five years, which peaked at 3.2 per 100,000 in the age group in March 2023 [11]. Fatality rates were similar to previous years across all age groups: 30% among patients ≥ 85 years and less than 5% among children under 5 years [11]. 

Winter months are also associated with a burden of GAS pharyngitis in people of all ages, but especially in children. Sore throat is one of the most common clinical problems in general practice, and the majority of them are viral, so most patients do not benefit from antibiotics. Clinical guidelines recommend prescribing antibiotics for pharyngitis only when there is a high probability that the condition has been caused by GAS [7,12].

Our study revealed that penicillin V, the first-choice antibiotic, was used in 41.0% of cases, followed by amoxicillin at 20.8% and cefadroxil at 12.5%. The high percentage of prescribed amoxicillin reveals poor adherence to the Polish guidelines limiting the use of a low dosage of amoxicillin to treat GAS pharyngitis [10]. Other prescribed antibiotics were clarithromycin, azithromycin, cefuroxime axetil, amoxicillin- clavulanate, levofloxacin, cefaclor, cefixime, and clindamycin. Notably, no strains resistant to penicillin were observed in Poland, reinforcing its efficacy and reliability. Moreover, 224 patients had negative rapid strep test results, but still received antibiotic treatment. It seems that doctors prescribe antibiotics out of fear that the patient could eventually develop complications. However, they do not remember that overprescribing is associated with the growing antibiotic resistance and side effects, e.g., diarrhea. Some patients might also expect a prescription for an antibiotic even if the rapid strep test result is negative. Another study indicated that 23% (80/345) of participants had a positive throat swab for GAS, but 65% (225/345) of all participants were prescribed immediate and 12% (43/345) delayed antibiotics [13]. Moreover, the authors of the mentioned study showed results of a retrospective analysis of the Centor score for 337 patients, of whom 49% had a low (score ≤ 2) and 48% had a high Centor score (>2) [13]. For 50% of participants, prescribing was in line with National Institute for Clinical Excellence (NICE) clinical guidelines, i.e., a Centor score of 3 or 4 [13]. The results of these studies indicated that antibiotics were overprescribed for sore throat, and this is worrying in light of global antimicrobial resistance.

The drug of choice, penicillin V, has proven highly effective when given at 500 mg twice daily for ten days. In patients with a penicillin allergy, macrolides, clindamycin, and first-generation cephalosporins can be used; however, first-generation cephalosporin should not be used in patients with immediate-type hypersensitivity to penicillin [7]. Group A strep bacteria with *Streptococcus pyogenes* are susceptible to penicillin or cephalosporins. Some studies have suggested greater efficacy with cephalosporins than penicillin, possibly because of their resistance to β-lactamase-producing organisms in the pharynx and due to their higher efficacy in killing ingested bacterial cells [14,15]. This means that cephalosporins are less likely to be affected by the enzymes produced by the bacteria that can degrade penicillin, making them more effective in some cases. However, cephalosporins are more expensive than penicillin, are associated with more significant side effects, and have a broader spectrum of activity. 

Nevertheless, some strains of *S. pyogenes* have developed resistance to other antibiotics. Azithromycin, clarithromycin, and erythromycin are effective in treating GAS pharyngitis. Macrolide antibiotics are an essential alternative to penicillin, especially in treating infections in people with penicillin hypersensitivity. Azithromycin is efficacious in treating GAS pharyngitis when given for only 5 days (500 mg on the first day and then 250 mg for 4 days taken as a single dose), clarithromycin for ten days (250 mg twice daily), and erythromycin for ten days (500 mg twice daily). However, macrolide-resistant GAS has been reported in many countries. Recent data indicated that macrolide-resistant strains of *S. pyogenes* were detected in Bulgaria (23–40%), Greece (20.4%), Spain (8.7%), Hungary (10.5%), Russia (12.1–17.2%), the USA (16–23%), Brazil (14.3%), China (94.74%), Japan (34.9–60%), Australia (6%), and northwest Ethiopia (21.4%) [16,17,18,19,20,21,22]. Also clindamycin-resistant strains of *S. pyogenes* are well known and vary geographically and temporally [7,22]. 

It is crucial to initiate penicillin treatment within nine days of the onset of symptoms of GAS pharyngitis, as it has been proven to be effective in preventing rheumatic fever. The absence of documented resistance of GAS to penicillin further underscores the importance of early treatment, highlighting its role in eradicating the organism from the pharynx. Treatment failures in GAS pharyngitis are a significant concern in preventing rheumatic fever. 

Our analysis showed no significant difference between positive rapid strep test results and patients’ sociodemographic data and comorbidities. Although we saw a relationship between negative rapid test results and diabetes, the difference between the groups did not meet the threshold for statistical significance. Given the small number of patients with diabetes, this observation is also at a very high risk of uncontrolled confounding. Moreover, some studies indicated for an increased risk of the GAS disease in patients with diabetes [23,24,25], and efforts to develop a vaccine and enhanced treatment regimens for iGAS might improve prognoses for patients with diabetes.

Limitations: Our study may not be representative for the whole population. Due to a limited sample size, results might differ in other regions. We did not analyze detailed clinical history,, e.g., allergy to antibiotics. Moreover, rapid strep tests have some limitations: they are not as accurate as molecular tests and have a higher rate of false negatives. We did not compare differences in management between adults and children. Furthermore, the doctors did not perform cultures and we had no information about the susceptibility pattern of *Streptococcus pyogenes* (especially the percentage of isolates resistant to macrolides).

## 5. Conclusions

The prevalence of GAS was 42.6% in the analyzed records of patients with pharyngitis, and 54.1% of all patients were prescribed antibiotics. Antibiotics were overprescribed for pharyngitis. Strategies are needed to promote rational antibiotic use. A system offering easy access to the monitoring of regional resistance patterns, their dynamics, and the spread of resistant strains is needed. 

## Figures and Tables

**Table 1:** Age of participants.

	N	Mean	Median	Minimum	Maximum	25Q	75Q	SD
Age [years]	1949	16.8	10.0	0.0	83.0	5.0	28.0	15.7

**Table 2:** Analysis of the time from symptom onset to rapid strep test.

	N	Mean	Median	Min.	Max.	25Q	75Q	SD
Time from symptom onset to rapid strep test	1637	2.67	2.00	0.00	30.00	1.00	3.00	2.23

**Table 3:** The distribution of antibiotics used for infections.

Antibiotic (Name of Active Substance)	No.	%
clarithromycin	46	4.4
penicillin V	431	41.0
azithromycin	33	3.1
amoxicillin	219	20.8
cefuroxime axetil	71	6.7
amoxicillin-clavulanate	90	8.6
levofloxacin	1	0.1
cefadroxil	131	12.5
cefaclor	11	1.0
cefixime	2	0.2
clindamycin	17	1.6

**Table 4:** The analysis of distribution of antibiotics and rapid strep test results.

Antibiotics	Negative Strep Test Result	Positive Strep Test Result
Clarithromycin	18	28
penicillin V	25	406
Azithromycin	22	11
Amoxicillin	78	141
cefuroxime axetil	24	47
amoxicillin-clavulanate	33	57
Levofloxacin	1	0
Cefadroxil	15	116
Cefaclor	3	8
Cefixime	2	0
Clindamycin	3	14

**Table 5:** Analysis of rapid strep test results and sociodemographic and clinical indicators.

Indicators:	Positive Strep Test Results (n)	Negative Strep Test Results (n)	*p* Value
Sex:			
Women	462	629	
Men	368	490	0.809
Age:			
0–18 years	579	735	
>18 years	251	384	0.0577
Vaccination status against influenza:			
No vaccination	670	927	
Vaccinated	65	63	
No data	95	129	0.151
Vaccination status against COVID-19:			
No vaccination	504	673	
Vaccinated	227	329	
No data	99	117	0.436
Diabetes:			
Yes	5	17	
No	825	1102	0.0582
Chronic respiratory diseases:			
Yes	42	62	
No	788	1057	0.641
Cardiovascular diseases:			
Yes	8	13	
No	822	1106	0.676
Hypertension:			
Yes	29	50	
No	801	1069	0.281
Congenital or acquired immunodeficiencies:			
Yes	5	7	
No	825	1112	0.948

## Data Availability

The data presented in this study are available on request from the corresponding author.
